# Multiple rod layers increase the speed and sensitivity of vision in nocturnal reef fishes

**DOI:** 10.1098/rspb.2023.1749

**Published:** 2023-11-22

**Authors:** Lily G. Fogg, Wen-Sung Chung, N. Justin Marshall, Fabio Cortesi, Fanny de Busserolles

**Affiliations:** ^1^ Queensland Brain Institute, The University of Queensland, Brisbane, Queensland 4072, Australia; ^2^ School of the Environment, The University of Queensland, Brisbane, Queensland 4072, Australia; ^3^ Zoological Institute, Department of Environmental Sciences, University of Basel, Basel, Switzerland

**Keywords:** coral reef fish, dim-light vision, fish ecology, physiology of vision, rod photoreceptor, visual adaptation

## Abstract

Most vertebrates have one layer of the dim-light active rod photoreceptors. However, multiple rod layers, known as a multibank retina, can be found in over 100 species of fish, including several deep-sea species and one family of nocturnally active reef fish, the Holocentridae. Although seemingly associated with increased photon catch, the function of multibank retinas remained unknown. We used an integrative approach, combining histology, electrophysiology and amino acid sequence analysis, applied to three species of nocturnal reef fishes, two holocentrids with a multibank retina (*Neoniphon sammara* and *Myripristis violacea*) and an apogonid with a single rod bank (*Ostorhinchus compressus*), to determine the sensory advantage of multiple rod layers. Our results showed that fish with multibank retinas have both faster vision and enhanced responses to bright- and dim-light intensities. Faster vision was indicated by higher flicker fusion frequencies during temporal resolution electroretinography as well as faster retinal release rates estimated from their rhodopsin proteins. Enhanced sensitivity was demonstrated by broadened intensity-response curves derived from luminous sensitivity electroretinography. Overall, our findings provide the first functional evidence for enhanced dim-light sensitivity using a multibank retina while also suggesting novel roles for the adaptation in enhancing bright-light sensitivity and the speed of vision.

## 1. Introduction

A diversity of visual adaptations has evolved across the animal kingdom to enhance function in a number of ecological niches. Due to the variability of light in their habitat, marine fishes possess the greatest diversity of visual adaptations among vertebrates [1–3]. To catch as many photons as possible, marine fishes living in dim-light environments such as in the deep sea or those active mainly at night show several scotopic adaptations. They include enlarged eyes or tubular eye structures [4,5], high expression of the rod opsin gene, *rh1* [6,7], high rod densities [8,9] and thick photoreceptor layers, either through longer rods or multiple layers of rods [7,10,11]. Although many of these adaptations have been attributed to increasing sensitivity, the function of multiple rod layers remains hypothetical [12,13].

A retina containing multiple layers of rods is known as a multibank retina and can contain 2–28 layers of stacked rods [11,14]. Multibank retinas have been found in representatives from at least 38 teleost fish families [3,15], the majority of which are deep-sea species [3]. Two predominant hypotheses have been suggested to explain their function. The first proposes that multibank retinas enhance luminous sensitivity by increasing the cumulative rod outer segment length available for photon capture [13]. The second suggests that they allow colour vision in dim light through spectral filtering at each layer and an opponent comparison between the layers [12]. Until now, few studies have examined the function of multibank retinas [16–18], due to the difficulty in accessing, handling and maintaining deep-sea fishes [11,19]. However, the recent characterization of multibank retinas in an easily accessible family of nocturnal coral reef fishes, Holocentridae [7], enabled us to test the sensitivity hypothesis.

The family Holocentridae is composed of two subfamilies: squirrelfishes (Holocentrinae) and soldierfishes (Myripristinae). They mainly inhabit shallow depth ranges, however, a few species dwell as deep as 640 metres [20,21]. Holocentrids are nocturnal [22] and as such, they have a typical dim light-adapted visual system with large eyes [4], a rod-dominated retina [7,23], a low focal ratio [10], a high summation of rods onto ganglion cells (GC) [24] and RH1 opsins with spectral sensitivities that are tuned to the dominant wavelengths at their prevalent depth [25]. They also possess a highly developed multibank retina, with up to 7 and 17 banks in squirrelfishes and soldierfishes, respectively [7]. However, holocentrids also show some photopic adaptations, including the potential for cone-mediated dichromatic colour vision [7]. Interestingly, they are most likely a group that has moved from the deep to a shallow-water habitat [26].

In this study, the sensitivity hypothesis for the function of the multibank retina was tested by assessing the visual systems of two species from the family Holocentridae (*Neoniphon sammara* and *Myripristis violacea,* from the subfamilies Holocentrinae and Myripristinae, respectively), and a non-multibank control species, another nocturnally active reef fish, *Ostorhinchus compressus* (family Apogonidae). Firstly, we examined retinal structure using histology. Then, we studied the luminous sensitivity and temporal resolution of their vision by recording the electrophysiological response of the whole eye to different light stimuli, using electroretinography (ERG) [27–31]. Finally, we estimated the retinal release rate of the rhodopsin proteins in each species (i.e. the time taken for the light-activated form of retinal, all-*trans* retinal, to be released from the opsin – a rate-limiting step in resetting of the phototransduction cascade) as a proxy for the speed of the regeneration of vision. Overall, our study sheds light on the unresolved function of an understudied visual adaptation in deep-sea and nocturnal coral reef fish as well as offering a broader insight into vision in vertebrates.

## 2. Methods

### (a) Animal collection and ethics

Details of all animals are given in electronic supplementary material, table S1. Adult fish were collected from the Great Barrier Reef around Lizard Island, Australia, or sourced from a supplier, Cairns Marine (https://www.cairnsmarine.com/), which also collects from the northern Great Barrier Reef. All collections and procedures were conducted under a Great Barrier Reef Marine Park Permit (G17/38160.1), a Queensland General Fisheries Permit (180731) and a University of Queensland's Animal Ethics Permit (QBI 304/16). Following euthanasia, all animals were photographed with a scale reference to quantify body length and eye diameter. Eyes were dissected and the eye cup preserved in RNAlater or paraformaldehyde (PFA; 4% (w/v) PFA in 0.01 M phosphate-buffered saline (PBS), pH 7.4) depending on the analyses.

### (b) Histology

**Figure 1.**
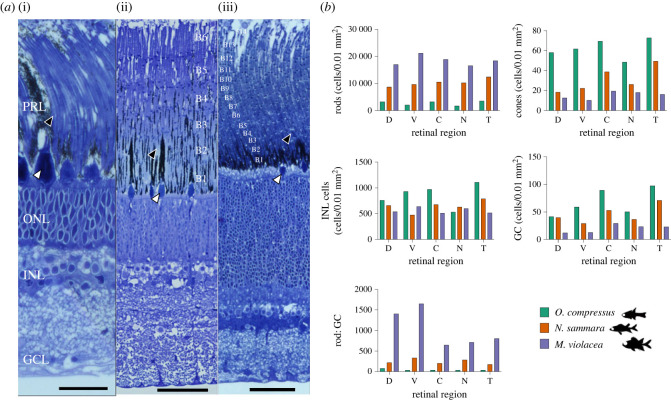
Retinal structure and cell densities*.* (*a*) Representative radial sections from the retina of (i) *O. compressus*, (ii) *N. sammara* and (iii) *M. violacea*. Rod banks are numbered as B_n_. Representative rod and cone outer segments are indicated by black and white arrows, respectively. (*b*) Densities of different types of retinal cells in five regions of the retina in *O. compressus* (*n* = 1), *N. sammara* (*n* = 1) and *M. violacea* (*n* = 1). PRL, photoreceptor layer; ONL, outer nuclear layer; INL, inner nuclear layer; GCL, ganglion cell layer; GC, ganglion cells; D, dorsal; V, ventral; C, central; N, nasal; T, temporal. Scale bars: 25 µm (ai), 50 µm (aii and aiii).

Five retinal regions (dorsal, ventral, central, nasal and temporal) were dissected, processed and sectioned from one PFA-fixed eye each for *O. compressus, N. sammara* and *M. violacea* as described previously [7]. The densities of key retinal cell types (i.e. cones, rods, inner nuclear layer (INL) cells and GC) per 0.01 mm^2^ of retina were estimated from sections using Fiji v1.53c [32] as described elsewhere (electronic supplemental material; [24]). Densities were corrected for cell size using Abercrombie's correction [33] (figure 1).

### (c) Electroretinography

Corneal electroretinography (ERG) recordings were conducted *in vivo* on whole, intact eyes to assess visual function using methods similar to those described in [29]. Fish were acclimatized to the recording chamber for 30 min, anaesthetized with 0.2 ml clove oil/litre seawater, immobilized with an intramuscular injection of 8.5 mg kg^−1^ gallamine triethiodide and ventilated with oxygenated seawater (electronic supplementary material, figure S1). After ≥40 min of dark adaptation, light stimuli were delivered to the eye using a custom-built, calibrated, broad-spectrum light source controlled via a PowerLab 4/26 DAQ module. Visual responses were detected through silver wire electrodes placed on the surface of the eye, amplified via a DP-103 amplifier and acquired in LabChart 8 v8.1.16. The system was grounded to the water of the recording chamber. Recordings were conducted at 28 ± 1°C at both day and night to control for any effects of temperature and circadian rhythm, respectively. Recordings were performed at the Lizard Island Research Station (LIRS) or the Queensland Brain Institute (QBI). Additional recordings were taken at both sites to compare results between the recording locations (electronic supplementary material, figure S2).

### (d) Temporal resolution electroretinography

**Figure 2.**
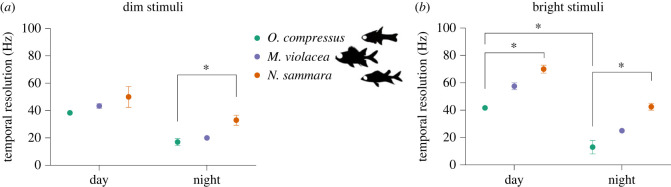
Temporal resolution electroretinography (ERG)*.* ERG waveforms were obtained for a range of stimulus frequencies from 5 to 95 Hz. The temporal correlation of resultant waveforms with the stimulus were used to derive the maximal temporal resolution (i.e. flicker fusion frequency) elicited using either (*a*) dim (4 lux) or (*b*) bright (384 lux) stimuli in *O. compressus* (*n* = 3 and 5 for day and night recordings, respectively), *N. sammara* (*n* = 3 and 5 for day and night recordings, respectively) and *M. violacea* (*n* = 3 and 5 for day and night recordings, respectively). Data represent mean ± s.e.m. Statistical significance (calculated from a Kruskal–Wallis with Dunn's multiple comparisons test): *, *p* < 0.05.

The temporal resolution of vision was assessed using flicker fusion frequency (FFF) ERGs on three or five individuals for day and night recordings, respectively, for *O. compressus, N. sammara* and *M. violacea*. FFF is the frequency at which retinal responses no longer follow the frequency of evenly spaced light pulses. Dark-adapted FFF ERGs were recorded by increasing the frequency of white light stimuli of constant intensity from 5 Hz to 95 Hz at increments of 5 Hz. Light pulses were 10 ms in duration and were repeated 30 times. Recordings were conducted for bright (384 lux) and dim (4 lux) stimuli (figure 2). The FFF threshold was determined either through visual inspection (at lower frequencies, less than 65 Hz) or by using the power spectrum to differentiate the signal and noise (at higher frequencies, ≥65 Hz) (electronic supplementary material; [30,34]). Statistics and graphs throughout the study were generated in GraphPad Prism v9.0.0.

### (e) Absolute sensitivity electroretinography

Absolute sensitivity ERGs were conducted for *N. sammara* (*n* = 4 for day recordings, *n* = 5 for night recordings), *M. violacea* (*n* = 4 for day and night) and *O. compressus* (*n* = 5 for day and night). The absolute (luminous) sensitivity of vision was determined using *V/logI* curves, which plot the normalized amplitude of the response, *V* (electronic supplementary material, figure S1), against the log of the intensity (*I*). These ERGs were recorded by increasing the intensity of a white light from 2.4 × 10^−8^ to 240 000 lux (i.e. −7.6 to 5.4 log_10_(lux)) in 0.3–0.6 log unit steps. Light stimuli were 100 ms pulses presented at 0.1–0.4 Hz (electronic supplementary material) and were repeated 10 times for each intensity. The mean response amplitudes were normalized to the maximal response (*V*_max_) and plotted against stimulus intensity to obtain the *V/logI* curve [29,35]. *V/logI* curves were used to calculate stimulus irradiances eliciting 5%, 50% (referred to as *K*_50_) and 95% of *V*_max_. *K*_50_ values are typically used to make interspecific comparisons of relative luminous sensitivity, while the dynamic range is a proxy for the breadth of visual functionality and was defined as the log_10_ irradiance range between 5% and 95% *V*_max_ [35,36]. All values were calculated separately for day and night experiments. To isolate the effect of the multibank retina, the V_max_-normalized responses were also normalized to eye diameter (as a proxy for retinal area, which is referred to as eye size) to obtain responses per unit of retina and analysed again as described above. To further understand how the visual response changed with intensity, representative ERG waveforms were analysed to obtain: (1) the time from stimulus presentation to the peak of the signal generated post-synaptic to the photoreceptors (i.e. time to b-wave peak; ms) and (2) the amplitude of the photoreceptor-derived peak (i.e. a-wave amplitude; mV). These values were obtained for dim (0.4 lux), moderate (125 lux) and bright (2165 lux for *O. compressus* and 5160 lux for *N. sammara* and *M. violacea*) stimuli, which matched the base, peak and decline of the *V/logI* curves, respectively.

### (f) Estimations of retinal release rate

Amino acid substitution sites involved in retinal release rate were analysed to estimate the retinal release time of the rhodopsin protein in each species (table 1). Firstly, 11 candidate amino acid (AA) substitution sites were identified from the literature [37–39]. Each substitution selected had a known effect on retinal release rate that has been previously calculated as the difference in retinal release half-life (*t_1/2_*; min) compared with a wild-type reference (either zebrafish [37], bovine [39] or catfish [40] rhodopsin, depending on the study). Notably, the effect on retinal release rate has not been characterized for all positively selected non-spectral substitutions in the literature (e.g. T97S in *N. sammara* and F116S and A164G in *M. violacea*) and that any substitutions that also affected spectral sensitivity were excluded from these analyses (i.e. residues 83, 122, 211, 261, 292 and 295) [41]. Next, the rod opsin coding sequences for *O. compressus* (MH979489.1)*, N. sammara* (MW219675.1) and *M. violacea* (MW219672.1) [6,7] were downloaded from GenBank (https://www.ncbi.nlm.nih.gov/genbank/) and translated into protein sequences. These were aligned and inspected for AA substitutions at each of the candidate sites in Geneious Prime v2021.1.1. Identified substitutions were used to estimate the cumulative change in retinal release (table 1). The absolute retinal release half-life was then calculated by subtracting the cumulative change from the half-life of wild-type bovine rhodopsin, which has previously been determined to be 13.9 min [37].

**Table 1.** Amino acid (AA) substitutions in nocturnal reef fishes linked to retinal release rates. Different AA substitutions in teleosts that have been found to have little effect on spectral tuning but alter retinal release rates [37–39] were examined in *O. compressus, N. sammara* and *M. violacea*. Each candidate AA substitution is given in the first column, and the corresponding AA found in the study species is given for each site. Substituted sites in the study species are in bold. The cumulative difference in retinal release *t_1/2_* (min) and the absolute retinal release *t_1/2_* (min) compared with wild-type bovine rhodopsin (13.9 min) [37] is given for each species.

AA substitution	*O. compressus*	*N. sammara*	*M. violacea*	Change in retinal release *t_1/2_*
I209V	T	F	**V**	−1.3
F213I	M	M	L	+1.5
V266L	C	**L**	**L**	+1.7
L290I	**I**	**I**	**I**	−1.3
V286I	V	L	L	−0.9
M123I	**I**	**I**	**I**	+4.9
G124A	**A**	S	G	+1.8
C165L	C	**L**	**L**	−0.9
V189I	**I**	**I**	**I**	+2.5
L59Q	L	L	L	−6.3
Y74F	Y	Y	Y	−1.9
**Cumulative change in retinal release *t_1/2_***	7.9	6.9	5.6	
**Retinal release *t_1/2_***	21.8	20.8	19.5	

## 3. Results

### (a) Holocentrids have high rod densities and high scotopic summation

Retinal architecture and cell densities were assessed in *O. compressus*, *N. sammara* and *M. violacea* (*n* = 1). All three species had duplex retinas composed of both rods and cones. However, while *O. compressus* only had a single layer of rods (figure 1*a*i, electronic supplementary material, figure S3), *N. sammara* and *M. violacea* had a maximum of 6 and 14 banks of rods, respectively (figure 1*a*ii–iii; electronic supplementary material, figure S3). The densities of all cell types were heterogeneous across the retina in all species (figure 1*b*; electronic supplementary material, table S2). The highest rod densities and summation of rods onto GC occurred in *M. violacea* (peak rod densities, 21 296 cells/0.01 mm^2^; peak rod:GC ratio, 1651.5 rods/GC) followed by *N. sammara* (peak rod, 12 403 cells/0.01 mm^2^; peak rod:GC, 332.6 rods/GC) and then *O. compressus* (peak rod, 3545 cells/0.01 mm^2^; peak rod:GC, 78.1 rods/GC). An inverse pattern was observed for cone and GC densities in all regions, with *O. compressus* having the highest densities and *M. violacea* the lowest (*O. compressus*: 72.8 cells/0.01 mm^2^ and 97.5 cells/0.01 mm^2^ for peak cone and GC, respectively; *N. sammara*: 49.4 cells/0.01 mm^2^ and 71.0 cells/0.01 mm^2^; *M. violacea*: 19.5 cells/0.01 mm^2^ and 29.2 cells/0.01 mm^2^). Finally, INL cell densities were also highest in *O. compressus* and lowest in *M. violacea* for most regions (i.e. dorsal, central and temporal) (peak INL*, O. compressus*: 1108 cells/0.01 mm^2^; *N. sammara*: 789 cells/0.01 mm^2^; *M. violacea*: 638 cells/0.01 mm^2^).

### (b) Holocentrids have a higher temporal resolution compared with the apogonid

Temporal resolution ERGs were conducted to determine the FFF (the point at which responses to evenly spaced light pulses can no longer be distinguished as separate) in response to dim (4 lux) and bright (384 lux) stimuli at day (*n* = 3) and night (*n* = 5) (electronic supplementary material, figure S1). Under all conditions, *N. sammara* attained the greatest FFF (mean ± s.e.m. at day and night, respectively: dim: 50 ± 7.6 Hz and 33 ± 3.7 Hz; bright: 70 ± 2.9 Hz and 42.5 ± 2.5 Hz; *p* < 0.05 except for dim stimuli during the day which was not significant (n.s.)), followed by *M. violacea* (dim: 43.3 ± 1.7 Hz and 20 ± 0 Hz; bright: 57.5 ± 2.5 Hz and 25 ± 0 Hz) and then *O. compressus* (dim: 38.3 ± 1.7 Hz and 17 ± 2.5 Hz; bright: 41.7 ± 1.7 Hz and 13 ± 4.9 Hz) (figure 2; electronic supplementary material, figure S4; table S3). Furthermore, holocentrids had lower FFFs when exposed to the dim stimulus compared with the bright stimulus at each time point (*p* < 0.05 for dim versus bright stimulus during the day and dim versus bright stimulus at night for both species; electronic supplementary material, table S3). However, the FFFs of *O. compressus* did not vary greatly with stimulus intensity. Finally, all species showed a trend towards lower FFFs at night compared with during the day, irrespective of stimulus intensity (*p* < 0.0001 for day versus night for bright stimulus and day versus night for dim stimulus for all species; electronic supplementary material, table S3)

### (c) Holocentrids have enhanced sensitivity compared with the apogonid to both bright and dim light at night

**Figure 3.**
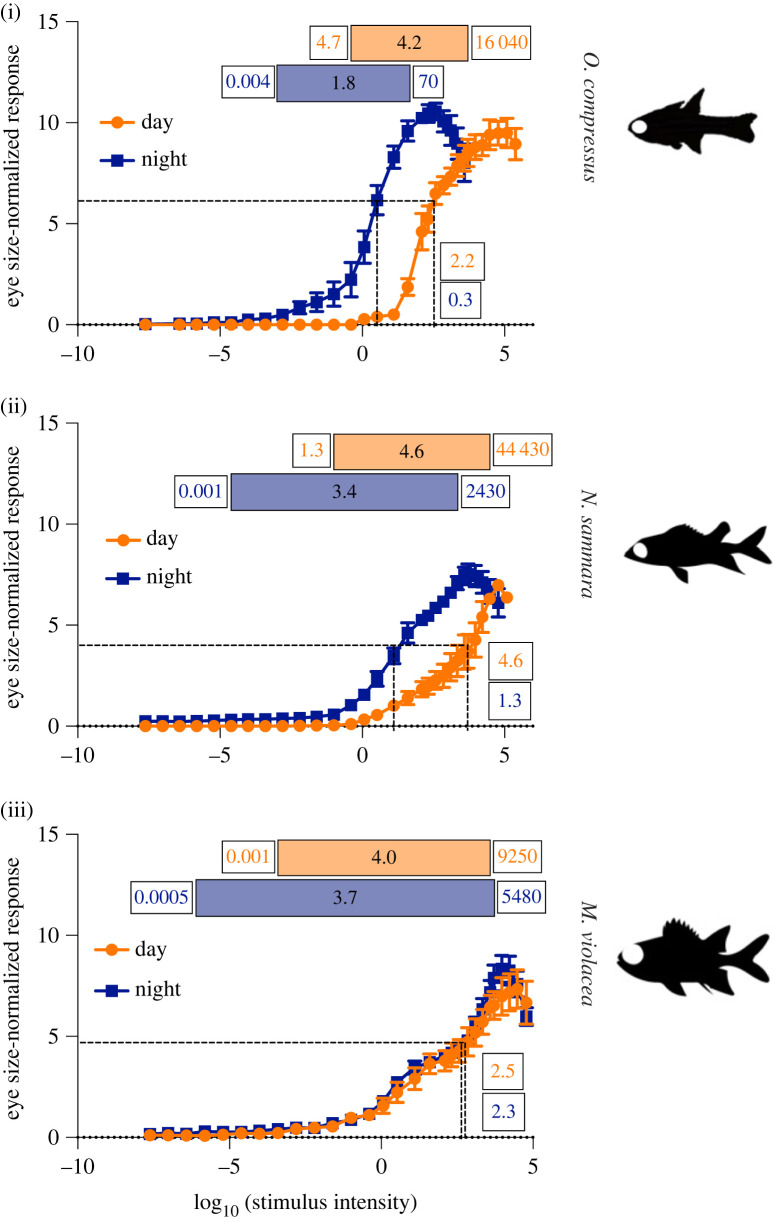
V/logI curves from absolute sensitivity electroretinography (ERG)*.* ERG waveforms were obtained for a range of intensities from 2.4 × 10^−8^ to 2.4 × 10^5^ lux (i.e. −7.6 to 5.4 log_10_(lux)). The mean b-wave amplitude from each set of waveforms was plotted against the log_10_ of the stimulus intensity (in lux) normalized to both the maximal response (V_max_; response given as % of V_max_) and eye size for (i) *O. compressus* (*n* = 5), (ii) *N. sammara* (*n* = 4 and 5 for day and night recordings, respectively) and (iii) *M. violacea* (*n* = 4) at day and night. Shaded boxes at the top represent each species' dynamic range (5 to 95% V_max_), numbers within shaded boxes represent its breadth (in log units), and numbers at the edges of the boxes represent the minimum (5% V_max_) and maximum values (95% V_max_) of the range (in lux). Dotted lines and adjacent numbers indicate K_50_ values (in log units). Orange and blue represent day and night experiments, respectively. Curve data are mean ± s.e.m.

Absolute sensitivity ERGs were recorded for *O. compressus* (*n* = 5), *N. sammara* (*n* = 4 and 5 for day and night recordings, respectively) and *M. violacea* (*n* = 4) during the day and night (electronic supplementary material, figure S1; figure S5). Firstly, *V/logI* curves were normalized to either *V*_max_ alone (for response of the entire eye; figure 3*a*) or *V*_max_ and eye size (for response per unit of retina; figure 3*b*). In all species, *V/logI* curves produced non-monotonic functions, with the amplitude of the b-wave representing the response post-synaptic to the photoreceptor, generally increasing with stimulus intensity until the maximal amplitude (*V*_max_) was reached, before subsequently decreasing due to bleaching. Notably, a subtle decrease in gradient occurred in the curves from *M. violacea* between stimulus intensities of approximately 40 and 700 lux (equivalent to 1.6–2.8 log_10_(lux)), before continuing to increase until the response reached its peak. A closer examination of the ERG waveforms themselves revealed that, in all species, the speed of the visual response (i.e. time taken for the b-wave to reach its peak) became faster at higher intensities (electronic supplementary material, figure S6). Additionally, the photoreceptor-derived component of the waveform (i.e. a-wave amplitude) also increased at higher intensities, very minimally in *O. compressus*, more substantially in *N. sammara* and greatly in *M. violacea* (electronic supplementary material, figure S6).

There were notable differences in the *V/logI* curves between diel period and species. The *V/logI* curves were bright-shifted during the day compared with the night for *O. compressus* and *N. sammara*, but not *M. violacea*. Furthermore, when considering the same diel period, the *V/logI* curves differed among the three species, with the nature of these differences quantified using 5% and 95% *V*_max_ values, the dynamic range and *K*_50_ values. Interspecific trends in these values were similar irrespective of whether the data was normalized to V_max_ alone (electronic supplementary material, figure S7) or *V*_max_ and eye size (figure 3). Notably, the K_50_ values were less useful than the other parameters measured in this study due to substantial interspecific differences in the shape of the *V/logI* curves at mesopic intensities. Therefore, only the 5% and 95% V_max_ values and the dynamic range have been interpreted here.

Both during the day and at night, *M. violacea* reached 5% *V*_max_ at the lowest intensity, followed by *N. sammara* and then *O. compressus* (figure 3), indicating that holocentrids could respond to dimmer intensities than *O. compressus*. Similarly, during the night, *M. violacea* reached 95% V_max_ at the highest intensity, followed by *N. sammara* and then *O.compressus*, indicating that the visual system of the holocentrids could also delay bleaching and continue to function at higher intensities than *O. compressus,* but only at night. By contrast, during the day, *N. sammara* reached 95% V_max_ at the highest intensity, followed by *O. compressus* and then *M. violacea*. As a result of the differences in 5% and 95% V_max_, *M. violacea* had the broadest dynamic range during the night, followed by *N. sammara* and then *O. compressus*. By contrast, *N. sammara* had the greatest dynamic range during the day, followed by *O. compressus* and then *M. violacea* (figure 3). Overall, these results indicate that at night, when rods are the dominantly active photoreceptor type, the holocentrids have superior visual functionality under both bright- and dim-light intensities than *O. compressus* but this is not the case during the day, when cones are the dominantly active photoreceptor type.

### (d) Holocentrids had faster estimated retinal release rates compared with the apogonid

The retinal release rates of each species' rhodopsin protein were estimated using AA substitutions. The *O. compressus* RH1 possessed four AA substitutions known to alter retinal release rate, while those in *N. sammara* and *M. violacea* had five and six AA substitutions, respectively (table 1). These substitutions resulted in increased estimated retinal release times for the rhodopsins of all three species when compared with wild-type rhodopsin. Estimations suggested that retinal release half-life was shortest in *M. violacea* (*t_1/2_* of 19.5 min), followed by *N. sammara* (20.8 min) and then *O. compressus* (21.8 min). Therefore, the rhodopsins of both holocentrids had faster estimated retinal release rates than *O. compressus*.

## 4. Discussion

Here, we investigated the retinal structure and visual function of nocturnal reef fishes with and without multibank retinas. Firstly, we confirmed that, at the morphological level, the three species investigated had visual systems that were well-adapted to their dim-light environments (figure 1; electronic supplementary material, figure S3; table S2). In accordance with their nocturnal lifestyle [6,7,42], all three species had high rod densities and high rod:GC summation, and low cone and GC densities compared with shallow-dwelling diurnal species [43–45]. Additionally, like other holocentrid species [7], *N. sammara* and *M. violacea* had multiple rod banks across the entire retina. Similar to other nocturnal reef fishes [6,7], all three species also retained some degree of photopic adaptation, with cones interspersed throughout the retina. However, the degree of scotopic and photopic adaptations varied among the three species with *N. sammara* and *M. violacea* showing greater adaptation for scotopic vision (i.e. higher rod densities and summation and multibank retinas) but inferior adaptation for photopic vision (i.e. lower cone densities) compared with *O. compressus*.

Secondly, this study examined temporal resolution (or speed) of vision in these fishes by determining the FFF (figure 2; electronic supplementary material, figure S4; table S3). Temporal resolution is fundamentally determined by the integration time of photoreceptors, with cones displaying faster dynamics than rods [46]. Thus, FFF is generally lower in conditions when rod responses dominate, such as in species with rod-only or rod-dominated retinas (e.g. deep-sea fishes), at night and for lower stimulus intensities [17,28]. Consequently, the maximal FFF of deeper-dwelling and nocturnal fishes ranges from about 9 to 40 Hz, compared with the 40 to 100 Hz in shallow-dwelling diurnal fishes [28,30,36]. Similar to findings in other fishes [47], the FFF of *O. compressus, N. sammara,* and *M. violacea* varied with diel period and stimulus intensity. All species had dim-stimulus night-time FFFs comparable to other nocturnal reef fishes, however, while the peak FFF (i.e. elicited with bright stimuli during the day) was within the range for other nocturnal fishes for *O. compressus* (approx. 40 Hz) [29], for both *N. sammara* (70 Hz) and *M. violacea* (approx. 60 Hz) the range shown was characteristic of diurnal fishes [29,30].

The fact that *O. compressus* had the highest cone and lowest rod densities but not the highest peak FFF implies that more complex neuronal mechanisms are at play in the holocentrids compared with the cardinalfish, likely due to the structure of the multibank retina. Indeed, the higher speed of the multibank retina could be related to photoreceptor size. High sensitivity requires a large membrane surface area, and this is often achieved through large photoreceptors. However, large photoreceptors may have slower activation rates than small photoreceptors (due to large cytosolic volume and therefore, slow ion concentration changes during activation) [48]. Theoretically, a retina with many layered, small photoreceptors, i.e. a multibank retina, could circumnavigate this trade-off between sensitivity and speed, potentially permitting the faster visual dynamics observed in the holocentrids. Notably, the only other multibank representative whose temporal resolution has been assessed was that of a deep-sea fish (*Lepidocybium flavobrunneum*) which had a much lower FFF (9 Hz; [17]) than the species examined in this study. This indicates that the speed of vision in a multibank retina is also influenced by the ecological demands of a species, since *L. flavobrunneum* leads a slow-moving life in the deep sea in contrast to the holocentrids which lead active lives in shallow waters [49,50]. Finally, the speed of vision is likely also affected by other physiological factors, such as the retinal release rate of each species’ rhodopsin proteins [51].

Finally, we assessed luminous sensitivity (figure 3; electronic supplementary material, figure S5). In fishes, luminous sensitivity usually varies with diel period due to a dominance of cone- and rod-based responses at day and night, respectively [28,46]. Our findings revealed that *N. sammara* and *O. compressus* were no exception, showing higher bright-light sensitivity during the day but higher dim-light sensitivity during the night. The sensitivity of *M. violacea* was, on the other hand, relatively constant. This indicates that *M. violacea* may only undergo a weak diel switch between photopic and scotopic systems. This is likely due to their lack of a well-developed photopic system to switch to, similar to some deep-sea fishes with pure rod retinas [52].

Luminous sensitivity also varies with retinal structure and ecology. For example, diurnal fish (with higher cone densities) have better daytime bright-light vision, while nocturnal fish (higher rod densities) have greater night-time dim-light visual capabilities [27,28]. Notably, the typical parameter used for interspecific comparisons of relative sensitivity, the K_50_ values [29], was not interpreted in this study due to interspecific differences in the *V/logI* curves at mesopic intensities. These differences may be due to variation in the rate of migration of the retinal pigment epithelium among the species [7] or differences in how they switch between the retinal circuits that process rod- versus cone-derived signals [53,54]. Regardless, visual function was still interpretable through the 5% and 95% *V*_max_ values and the dynamic range. Using these values, this study found stronger dim-light visual responses both during the day and at night (i.e. dim-shifted 5% V_max_ values) with increasing rod densities and rod banking. This supports the idea that the multibank retina enhances dim-light vision.

Enhanced bright-light vision (i.e. bright-shifted 95% *V*_max_ values) was also correlated with increasing rod densities and banking (and decreasing cone densities), but only during the night, when rods are the dominantly active photoreceptor type. Bright-light vision is usually cone mediated, however, in some species with rod-dominated retinas and few cones, e.g. mice, rods have been shown to function under bright-light conditions [55,56]. Our data suggest that the holocentrids may also use rods for bright-light vision. It is unlikely that holocentrids need to respond to bright intensities at night and instead, the rods are likely to be facilitating bright-light vision simply when a species has very few cones available for this purpose (e.g. when the retina is rod dominated in dim-light specialized species). Since this did not occur during the day (i.e. *O. compressus* had higher daytime 95% *V*_max_ values than *M. violacea*), our data also suggest that the rods have less involvement in photopic vision when cones can be used instead (e.g. when the retina has higher cone densities). However, the photopic visual capabilities of the holocentrids would likely still be sufficient to meet their daytime ecological demands, such as courtship and predator avoidance [57,58]. Overall, holocentrids seem to have an enhanced capacity to regenerate the visual response which permits some potentially rod-based vision under brighter intensities, likely enhancing achromatic contrast sensitivity. This finding suggests that, as previously proposed [59], holocentrids use the different layers of rods, and therefore, higher numbers of outer segments, to increase chromophore recycling.

Our study suggests that the rods in the holocentrid multibank retina can still function at brighter intensities. However, rhodopsin normally bleaches at high intensities. A key reason for this bleaching is the slower retinal release rate of rhodopsin compared with the cone opsins, and therefore, slower resetting of the phototransduction cascade [48,60]. Amino acid-based estimations of retinal release rates in our study species revealed that the holocentrids may have accelerated retinal release rates compared with cardinalfishes, which would allow their rods to recover more rapidly post-bleaching (table 1). Supporting a faster recovery rate in holocentrids, we also found higher temporal resolution at both day and night compared with *O. compressus* despite their less well-developed photopic visual systems. Furthermore, work in mice has shown that rods can recover and respond to bright intensities and that this is facilitated by more efficient post-bleaching regeneration at higher light intensities compared with lower intensities [55,56]. Future work using *in vitro* regeneration experiments to test the retinal release rates of holocentrid RH1 visual pigments may be used to explain how their rods continue to function at brighter intensities, similar to cones.

Overall, our findings suggest a dual role for the holocentrid multibank retina, where at dim intensities it functions to enhance photon capture while at bright intensities, it functions to regenerate the visual response in a rod-dominated system, allowing the eye to function at both lower and higher intensities than a retina with a single rod bank. Interestingly, these dual rod- and cone-like physiological properties of the rods in the holocentrids are reminiscent of findings in skates [61,62]. Skates have a pure rod retina; however, their rods exhibit physiological properties of both photoreceptor types. Specifically, at dimmer intensities, skate rods show rod-like physiology, while at higher intensities, they show cone-like physiological properties, including faster photoreceptor responses and visual responses even under very bright illumination [61]. Furthermore, the transformation between rod- and cone-like function is a slow process in the skates, similar to the delayed switch observed in the holocentrids, particularly *M. violacea*. The dual functionality of the skate retina was hypothesized to be due to the fact that they are dominantly but not exclusively nocturnal [62], also similar to the holocentrids. Although intriguing, the mechanism that underlies the dual functionality of the holocentrid retina remains to be determined. Since cone-like synaptic terminals are thought to contribute to this physiological phenomenon in skates [61,63], it may be worth examining the ultrastructure of the photoreceptor synapses in the holocentrids in future.

Irrespective of the underlying mechanism, enhanced visual functionality at both bright- and dim-light intensities aligns well with the ecology of holocentrids, since they are nocturnal foragers but are still somewhat active on the reef during the day [57]. Overall, our results strongly support one of the predominant hypotheses on the function of the multibank retina [11]. However, it still remains possible that the multibank retina also permits colour vision in dim light [12]. This second possibility is currently under behavioural investigation using the relatively accessible and easy-to-maintain holocentrid species.

## Data Availability

Most of the data are included in the main text and/or supporting information. Raw data for electrophysiological recordings are publicly available on Dryad (https://doi.org/10.5061/dryad.280gb5mtf [64]). Data sources: the published rhodopsin sequences used in the study are cited in the Materials and Methods. All other data are original. Supplementary material is available online [65].

## References

[1] Cohen JE, Beddington JR, Cushing DH, May RM, Steele JH. 1994 Marine and continental food webs: three paradoxes? Phil. Trans. R. Soc. Lond. B **343**, 57-69. (10.1098/rstb.1994.0008)

[2] Cortesi F, Mitchell LJ, Tettamanti V, Fogg LG, de Busserolles F, Cheney KL, Marshall NJ. 2020 Visual system diversity in coral reef fishes. Semin. Cell Dev. Biol. **106**, 31-42. (10.1016/j.semcdb.2020.06.007) 32593517

[3] de Busserolles F, Fogg L, Cortesi F, Marshall J. 2020 The exceptional diversity of visual adaptations in deep-sea teleost fishes. Semin. Cell Dev. Biol. **106**, 20-30. (10.1016/j.semcdb.2020.05.027) 32536437

[4] Schmitz L, Wainwright PC. 2011 Nocturnality constrains morphological and functional diversity in the eyes of reef fishes. BMC Evol. Biol. **11**, 338. (10.1186/1471-2148-11-338) 22098687 PMC3240680

[5] Collin SP, Hoskins RV, Partridge JC. 1997 Tubular eyes of deep-sea fishes: a comparative study of retinal topography (Part 1 of 2). Brain Behav. Evol. **50**, 335-346. (10.1159/000113345) 9406644

[6] Luehrmann M, Carleton KL, Cortesi F, Cheney KL, Marshall NJ. 2019 Cardinalfishes (Apogonidae) show visual system adaptations typical of nocturnally and diurnally active fish. Mol. Ecol. **28**, 3025-3041. (10.1111/mec.15102) 30977927

[7] de Busserolles F, Cortesi F, Fogg L, Stieb SM, Luehrmann M, Marshall NJ. 2021 The visual ecology of Holocentridae, a nocturnal coral reef fish family with a deep-sea-like multibank retina. J. Exp. Biol. **224**, jeb233098. (10.1242/jeb.233098) 33234682

[8] Pankhurst NW. 1989 The relationship of ocular morphology to feeding modes and activity periods in shallow marine teleosts from New Zealand. Environ. Biol. Fishes **26**, 201-211. (10.1007/BF00004816)

[9] Shand J. 1994 Changes in the visual system of teleost fishes during growth and settlement: an ecological perspective. PhD thesis, James Cook University, Australia.

[10] McFarland WN. 1991 The visual world of coral reef fishes. In The ecology of fishes on coral reefs (ed. PF Sale), pp. 16-36. San Diego, CA: Academic Press, Inc.

[11] Wagner HJ, Frohlich E, Negishi K, Collin SP. 1998 The eyes of deep-sea fish. II. Functional morphology of the retina. Prog. Retinal Eye Res. **17**, 637-685. (10.1016/S1350-9462(98)00003-2) 9777652

[12] Denton EJ, Locket NA. 1989 Possible wavelength discrimination by multibank retinae in deep-sea fishes. J. Mar. Biol. Assoc. UK **69**, 409-435. (10.1017/S0025315400029507)

[13] Frohlich E, Wagner HJ. 1998 Development of multibank rod retinae in deep-sea fishes. Vis. Neurosci. **15**, 477-483. (10.1017/S095252389815304X) 9685200

[14] Locket NA. 1985 The multiple bank rod fovea of *Bajacalifornia drakei*, an alepocephalid deep-sea teleost. Phil. Trans. R. Soc. Lond. B **224**, 7-22. (10.1098/rspb.1985.0018)

[15] Awaiwanont K, Gunarso W, Sameshima M, Hayashi S, Kawamura G. 2001 Grouped, stacked rods and tapeta lucida in the retina of Japanese anchovy *Engraulis japonicus*. Fisheries Science. **67**, 804-810. (10.1046/j.1444-2906.2001.00326.x)

[16] Meyer-Rochow VB, Coddington PA. 2003 Eyes and vision of the New Zealand Torrentfish *Cheimarrichthys fosterae* Von Haast (1874): histology, photochemistry and electrophysiology. In Fish adaptations (eds AL Val, BG Kapoor), pp. 337-383. Enfield, New Hampshire & Plymouth: Oxford and IBH Publ. & M/s Sci. Publ.

[17] Landgren E, Fritsches K, Brill R, Warrant E. 2014 The visual ecology of a deep-sea fish, the escolar *Lepidocybium flavobrunneum* (Smith, 1843). Philos. Trans. R. Soc. Lond. B **369**, 20130039. (10.1098/rstb.2013.0039) 24395966 PMC3886327

[18] Shapley R, Gordon J. 1980 The visual sensitivity of the retina of the conger eel. Phil. Trans. R. Soc. Lond. B **209**, 317-330. 10.1098/rspb.1980.0097 6107919

[19] Hess M, Melzer RR, Smola U. 1998 The photoreceptors of *Muraena helena* and *Ariosoma balearicum* - a comparison of multiple bank retinae in anguilliform eels (Teleostei). Zoologischer Anzeiger. **237**, 127-137.

[20] Nelson JS. 1994 Fishes of the world, 3rd edn. New York, NY: John Wiley & Sons, Inc.

[21] Greenfield DW, Randall JE, Psomadakis PN. 2017 A review of the soldierfish genus *Ostichthys* (Beryciformes: Holocentridae), with descriptions of two new species from Myanmar. Journal of the Ocean Science Foundation. **26**, 1-33.

[22] Horn MH, Karen LM, Chotkowski MA. 1999 Intertidal fishes: life in two worlds, p. 399. Academic Press.

[23] Fogg LG, Cortesi F, Lecchini D, Gache C, Marshall NJ, de Busserolles F. 2022 Development of dim-light vision in the nocturnal reef fish family Holocentridae I: retinal gene expression. J. Exp. Biol. **225**, jeb244513. (10.1242/jeb.244513) 35929500 PMC9482368

[24] Fogg LG, Cortesi F, Lecchini D, Gache C, Marshall NJ, de Busserolles F. 2022 Development of dim-light vision in the nocturnal reef fish family Holocentridae II: retinal morphology. J. Exp. Biol. **225**, jeb244740. (10.1242/jeb.244740) 35929495 PMC9482369

[25] Toller W. 1996 Rhodopsin evolution in the Holocentridae (Pisces: Beryciformes). PhD thesis, University of Southern California, USA.

[26] Yokoyama S, Tada T, Zhang H, Britt L. 2008 Elucidation of phenotypic adaptations: molecular analyses of dim-light vision proteins in vertebrates. Proc. Natl Acad. Sci. USA **105**, 13 480-13 485. (10.1073/pnas.0802426105) 18768804 PMC2533215

[27] Kobayashi H. 1962 A comparative study on electroretinogram in fish, with special reference to ecological aspects. Shimonoseki College of Fisheries **353**, 407-538.

[28] Ali MA. 1975 Vision in fishes: new approaches in research, p. 824. New York and London: Plenum Publishing Corporation.

[29] Horodysky AZ, Brill RW, Warrant EJ, Musick JA, Latour RJ. 2008 Comparative visual function in five sciaenid fishes inhabiting Chesapeake Bay. J. Exp. Biol. **211**, 3601. (10.1242/jeb.023358) 18978225

[30] Chung WS, Marshall NJ, Watson SA, Munday PL, Nilsson GE. 2014 Ocean acidification slows retinal function in a damselfish through interference with GABAA receptors. J. Exp. Biol. **217**, 323-326. (10.1242/jeb.092478) 24477607

[31] Gao X, Lin S, Zhang M, Lyu M, Liu Y, Luo X, You W, Ke C. 2022 Review: use of electrophysiological techniques to study visual functions of aquatic organisms. Frontiers in Physiology **13**, 52. (10.3389/fphys.2022.798382) PMC8829447 35153830

[32] Schindelin J et al. 2012 Fiji: an open-source platform for biological-image analysis. Nat. Methods **9**, 676. (10.1038/nmeth.2019) 22743772 PMC3855844

[33] Abercrombie M. 1946 Estimation of nuclear population from microtome sections. The Anatomical Record **94**, 239-247. (10.1002/ar.1090940210) 21015608

[34] Fritsches KA, Brill RW, Warrant EJ. 2005 Warm eyes provide superior vision in swordfishes. Current biology: CB. **15**, 55-58. (10.1016/j.cub.2004.12.064) 15649365

[35] Frank TM. 2003 Effects of light adaptation on the temporal resolution of deep-sea crustaceans. Integr. Comp. Biol. **43**, 559-570. (10.1093/icb/43.4.559) 21680464

[36] Horodysky AZ, Brill RW, Crawford KC, Seagroves ES, Johnson AK. 2013 Comparative visual ecophysiology of mid-Atlantic temperate reef fishes. Biology Open **2**, 1371. (10.1242/bio.20136825) 24285711 PMC3863422

[37] Morrow JM, Chang BSW. 2015 Comparative mutagenesis studies of retinal release in light-activated zebrafish rhodopsin using fluorescence spectroscopy. Biochemistry **54**, 4507-4518. (10.1021/bi501377b) 26098991

[38] Castiglione GM, Schott RK, Hauser FE, Chang BSW. 2017 Convergent selection pressures drive the evolution of rhodopsin kinetics at high altitudes via nonparallel mechanisms. Evolution **72**, 170-186. (10.1111/evo.13396) 29143302

[39] Hauser FE, Ilves KL, Schott RK, Castiglione GM, Lopez-Fernandez H, Chang BSW. 2017 Accelerated evolution and functional divergence of the dim light visual pigment accompanies cichlid colonization of Central America. Mol. Biol. Evol. **34**, 2650-2664. (10.1093/molbev/msx192) 28957507

[40] Castiglione GM et al. 2017 Evolution of nonspectral rhodopsin function at high altitudes. Proc. Natl Acad. Sci. USA **114**, 7385-7390. (10.1073/pnas.1705765114) 28642345 PMC5514753

[41] Yokoyama S, Takenaka N. 2004 The molecular basis of adaptive evolution of squirrelfish rhodopsins. Mol. Biol. Evol. **21**, 2071-2078. (10.1093/molbev/msh217) 15269277

[42] Fishelson L, Ayalon G, Zverdling A, Holzman R. 2004 Comparative morphology of the eye (with particular attention to the retina) in various species of cardinal fish (Apogonidae, Teleostei). Anat. Rec. A, Discov. Mol. Cell. Evol. Biol. **277**, 249-261. (10.1002/ar.a.20005) 15052652

[43] Shand J. 1997 Ontogenetic changes in retinal structure and visual acuity: a comparative study of coral-reef teleosts with differing post-settlement lifestyles. Environ. Biol. Fishes. **49**, 307-322. (10.1023/A:1007353003066)

[44] Ahlbert I-B. 1976 Organization of the cone cells in the retinae of Salmon (*Salmo salar*) and Trout (*Salmo trutta trutta*) in relation to their feeding habits. Acta Zoologica. **57**, 13-35. (10.1111/j.1463-6395.1976.tb00208.x)

[45] Shand J. 1994 Changes in retinal structure during development and settlement of the goatfish *Upeneus tragula*. Brain Behav. Evol. **43**, 51-60. (10.1159/000113624) 8306191

[46] Perlman I. 2001 The Electroretinogram: ERG. In Webvision: The organization of the retina and visual system (eds H Kolb, E Fernandez, R Nelson). Salt Lake City: University of Utah Health Sciences Center. 21413389

[47] Horodysky AZ, Brill RW, Warrant EJ, Musick JA, Latour RJ. 2010 Comparative visual function in four piscivorous fishes inhabiting Chesapeake Bay. J. Exp. Biol. **213**, 1751-1761. (10.1242/jeb.038117) 20435826

[48] Ingram NT, Sampath AP, Fain GL. 2016 Why are rods more sensitive than cones? J. Physiol. **594**, 5415-5426. (10.1113/JP272556) 27218707 PMC5043029

[49] Gladfelter WB, Johnson WS. 1983 Feeding niche separation in a guild of tropical reef fishes (Holocentridae). Ecology **64**, 552-563. (10.2307/1939975)

[50] Greenfield DW. 2002 Holocentridae: Squirrelfishes (Soldierfishes). In The living marine resources of the western central Atlantic. FAO species identification guide for fishery purposes and American society of ichthyologists and herpetologists special publication, vol. 5 (ed. KE Carpenter), pp. 1192-1202. Rome, Italy: Food and Agriculture Organization of the United Nations.

[51] Castiglione GM, Chang BS. 2018 Functional trade-offs and environmental variation shaped ancient trajectories in the evolution of dim-light vision. Elife **7**, e35957. (10.7554/eLife.35957) 30362942 PMC6203435

[52] Blaxter JHS, Staines M. 1970 Pure-cone retinae and retinomotor responses in larval teleosts. J. Mar. Biol. Assoc. UK **50**, 449-464. (10.1017/S0025315400004641)

[53] Demb JB, Singer JH. 2015 Functional circuitry of the retina. Annu. Rev. Vision Sci. **1**, 263-289. (10.1146/annurev-vision-082114-035334) PMC5749398 28532365

[54] Dunn FA, Doan T, Sampath AP, Rieke F. 2006 Controlling the gain of rod-mediated signals in the Mammalian retina. J. Neurosci. **26**, 3959-3970. (10.1523/JNEUROSCI.5148-05.2006) 16611812 PMC6673884

[55] Kelber A. 2018 Vision: rods see in bright light. Curr. Biol. **28**, R364-R366. (10.1016/j.cub.2018.02.062) 29689214

[56] Tikidji-Hamburyan A et al. 2017 Rods progressively escape saturation to drive visual responses in daylight conditions. Nat. Commun. **8**, 1813. (10.1038/s41467-017-01816-6) 29180667 PMC5703729

[57] Winn H, Marshall JA, Hazlett B. 1964 Behavior, diel activities, and stimuli that elicit sound production and reactions to sounds in the Longspine Squirrelfish. Copeia. **1964**, 413-425. (10.2307/1441036)

[58] Carlson BA, Bass AH. 2000 Sonic/vocal motor pathways in Squirrelfish (Teleostei, Holocentridae). Brain Behav. Evol. **56**, 14-28. (10.1159/000006674) 11025341

[59] Frohlich E, Wagner H-J. 1996 Rod outer segment renewal in the retinae of deep-sea fish. Vision Res. **36**, 3183-3194. (10.1016/0042-6989(96)00025-9) 8917778

[60] Chen M-H, Kuemmel C, Birge RR, Knox BE. 2012 Rapid release of retinal from a cone visual pigment following photoactivation. Biochemistry **51**, 4117-4125. (10.1021/bi201522h) 22217337 PMC3607377

[61] Dowling JE, Ripps H. 1990 On the duplex nature of the skate retina. J. Exp. Zool. **256**, 55-65. (10.1002/jez.1402560509) 1982496

[62] Ripps H, Dowling JE. 1990 Structural features and adaptive properties of photoreceptors in the skate retina. J. Exp. Zool. **256**, 46-54. (10.1002/jez.1402560508) 1982495

[63] Magaña-Hernandez L et al. 2023 The functionally plastic rod photoreceptors in the simplex retina of Little skate (*Leucoraja erinacea*) exhibit a hybrid rod-cone morphology and enhanced synaptic connectivity. See https://www.biorxiv.org/content/10.1101/2023.06.28.546621v1 (10.1101/2023.06.28.546621).

[64] Fogg LG, Chung W-S, Justin Marshall N, Cortesi F, de Busserolles F. 2022 Data from: Multiple rod layers increase the speed and sensitivity of vision in nocturnal reef fishes. Dryad Digital Repository. (10.5061/dryad.280gb5mtf) PMC10688437 37989239

[65] Fogg LG, Chung W-S, Justin Marshall N, Cortesi F, de Busserolles F. 2023 Multiple rod layers increase the speed and sensitivity of vision in nocturnal reef fishes. Figshare. (10.6084/m9.figshare.c.6917874) PMC10688437 37989239

